# Respiratory Syncytial Virus Vaccination as Cardiopulmonary and Cardiovascular Risk Mitigation in Adults with Heart Disease

**DOI:** 10.3390/jcm15176602

**Published:** 2026-08-26

**Authors:** Clara Bonanad, Vivencio Barrios, Guillermo Barreres, Nora Garcia-Collado, Daniela Maidana, David Vivas, Sergio Raposeiras, Elena Fortuny, Diego Segura-Rodriguez, Gonzalo Alonso-Salinas, Esther Redondo, Alberto Garcia-Lledó, Sergio García-Blas

**Affiliations:** 1 Department of Medicine, University of Valencia, 46010 Valencia, Spain; 2 Fundación Para la Investigación del Hospital Clínico de la Comunidad Valenciana (INCLIVA), 46010 Valencia, Spain; gbarreres@incliva.es (G.B.); nora.garcia.collado@gmail.com (N.G.-C.); 3 Centro de Investigación Biomédica en Red de Enfermedades Cardiovasculares (CIBERCV), Instituto de Salud Carlos III, 28029 Madrid, Spain; 4 Department of Cardiology, Hospital Clínico Universitario de Valencia, 46010 Valencia, Spain; 5 Department of Cardiology, Hospital Universitario Ramón y Cajal, Universidad de Alcalá, 28034 Madrid, Spain; vivenciobarrios@gmail.com; 6 Department of Cardiovascular Surgery, Hospital Universitario Virgen Macarena, 41042 Sevilla, Spain; danielamaidana@campus.fmed.uba.ar; 7 Department of Cardiology, Hospital Clínico San Carlos, 28040 Madrid, Spain; dvivas@secardiologia.es; 8 Department of Cardiology, Hospital Universitario Álvaro Cunqueiro, 38312 Vigo, Spain; raposeiras26@hotmail.com; 9 Department of Cardiology, Hospital Universitario Son Espases, 07120 Palma de Mallorca, Spain; elena.fortuny@ssib.es; 10 Department of Cardiology, Hospital Universitario Clínico San Cecilio, 18016 Granada, Spain; diegoseguracardio@gmail.com; 11 Instituto de Investigación Biosanitaria ibs.GRANADA, 18012 Granada, Spain; 12 Department of Medicine, University of Granada, 18071 Granada, Spain; 13 Department of Cardiology, Hospital Universitario de Navarra, 31008 Pamplona, Spain; gonzalol.alonso@gmail.com; 14 Departamento de Ciencias de la Salud, Universidad Pública de Navarra (UPNA), 31006 Pamplona, Spain; 15 Technical Unit for Vaccination and International Health, Madrid Salud Autonomous Organization, 28007 Madrid, Spain; redondome@madrid.es; 16 Departamento de Cardiología, Hospital Universitario Príncipe de Asturias, 28805 Alcala de Henares, Spain; alberto.garcia-lledo@uah.es

**Keywords:** respiratory syncytial virus, respiratory syncytial virus vaccination, cardiovascular disease, heart failure, older adults

## Abstract

**Background/Objectives:** Respiratory syncytial virus (RSV) causes morbidity in older adults with cardiovascular disease, in whom infection may precipitate severe lower respiratory tract disease, hospitalization, functional decline, and cardiovascular destabilization. We evaluate the evidence for RSV vaccination in cardiovascular disease and propose a jurisdiction-adaptable framework for integrating vaccination into cardiovascular care. **Methods:** Observational studies, randomized trials, pragmatic and test-negative studies, prespecified cardiovascular analyses of DAN-RSV, and eligibility recommendations were narratively synthesized and translated into a pathway encompassing patient identification, eligibility and risk assessment, vaccine delivery and co-administration, documentation, and follow-up. **Results:** Acute cardiac events are frequent during RSV hospitalization, and cardiovascular risk may persist after infection. Randomized trials establish protection against RSV-associated lower respiratory tract disease and respiratory illness, while real-world studies support effectiveness against RSV-related hospitalization. DAN-RSV reduced all-cause cardiorespiratory hospitalization but did not demonstrate significant reductions in cardiovascular hospitalization, myocardial infarction, heart failure (HF) hospitalization, atrial fibrillation, stroke, cardiovascular death, or major adverse cardiovascular events; observational associations with lower post-vaccination cardiovascular-event rates remain hypothesis-generating. The framework prioritizes adults aged ≥75 years and those aged 50–74 years with high-risk cardiovascular conditions where consistent with national recommendations, embeds vaccination assessment across cardiology, HF, primary care, pharmacy, and long-term care, and incorporates co-administration, documentation, and outcome surveillance. **Conclusions:** The principal contribution is a pathway for moving RSV vaccination from recommendation to routine preventive care for adults with cardiovascular disease. It anchors implementation in established RSV and cardiorespiratory benefits while treating cardiovascular-event reduction as a research priority rather than a demonstrated indication.

## 1. Introduction

Traditionally, cardiovascular prevention has been based on lipid-lowering therapy, blood pressure control, antithrombotic treatment, smoking cessation, diabetes management, physical exercise and the treatment of heart failure, in accordance with clinical guidelines. However, infections can be clinically significant destabilizing factors in cardiovascular disease. Influenza has the strongest historical evidence in this regard, but Respiratory Syncytial Virus (RSV) has rapidly become a major issue in adult vaccination, as it causes considerable morbidity in older people and in patients with chronic cardiopulmonary diseases [1,2,3,4,5,6,7].

RSV infection in adults can range from mild upper respiratory tract symptoms to severe lower respiratory tract disease, pneumonia, respiratory failure and hospitalization. Patients with chronic cardiovascular disease are vulnerable because RSV can increase oxygen demand, impair oxygen delivery, worsen pulmonary pressures and trigger inflammation and hemodynamic stress. In heart failure, even a mild respiratory insult can lead to congestion, arrhythmia, renal dysfunction and hospital readmission [1,5].

The availability of adult RSV vaccines has changed the practical question for cardiologists. It is no longer sufficient to ask whether RSV is a respiratory pathogen; clinicians must ask which cardiovascular patients are eligible for vaccination, what outcomes vaccination has been shown to prevent, and what cardiovascular claims are justified. This distinction is central. RSV vaccination is well supported for prevention of RSV-associated respiratory disease in older adults [8,9,10,11,12]; direct prevention of major adverse cardiovascular events (MACE) remains an active area of research rather than an established conclusion equivalent to the influenza-vaccine literature [13,14,15,16,17,18,19].

This review therefore uses cautious terminology. For influenza, some authors appropriately discuss vaccination as cardiovascular prevention in high-risk secondary-prevention populations. For RSV, the current evidence supports the more precise framing of cardiopulmonary and cardiovascular risk mitigation: preventing RSV disease and severe hospitalization in eligible adults with heart disease while acknowledging that direct MACE and cardiovascular-mortality effects have not yet been conclusively demonstrated. Several authors have already highlighted flu vaccination as a cardiovascular prevention measure in high-risk patients undergoing secondary prevention.

## 2. Objectives

This narrative review has five main objectives: first, to summarize the evidence linking RSV infection with acute and longer-term cardiovascular events in adults; second, to review efficacy and effectiveness data for adult RSV vaccines; third, to examine the emerging evidence on cardiorespiratory and cardiovascular outcomes after RSV vaccination; fourth, to summarize current adult recommendations relevant to patients with cardiovascular disease; and fifth, to translate the evidence into practical implementation pathways for cardiology, heart failure care, cardiac rehabilitation, primary care, pharmacy, long-term care, and public health.

The review intentionally separates four evidentiary questions that are often conflated: (1) whether RSV infection is associated with cardiovascular events; (2) whether RSV vaccines prevent RSV-associated respiratory disease; (3) whether RSV vaccines prevent RSV-associated hospitalization; and (4) whether RSV vaccines reduce cardiovascular endpoints such as myocardial infarction, stroke, heart failure hospitalization, atrial fibrillation, cardiovascular death, or MACE. These questions require different designs and should not be interpreted as numerically interchangeable.

## 3. Literature Search and Evidence Selection

This narrative review was informed by structured electronic searches of PubMed/MEDLINE, the Cochrane Library, and ClinicalTrials.gov from database inception to 1 July 2026. Regulatory and public-health sources, including [specify the organizations searched, e.g., the European Medicines Agency, the US Food and Drug Administration, the Centers for Disease Control and Prevention/Advisory Committee on Immunization Practices, and the UK Health Security Agency], were searched through the same date. Search concepts encompassed RSV infection, adult RSV vaccination, cardiovascular disease and heart failure, respiratory and cardiovascular outcomes, vaccine efficacy and effectiveness, safety, co-administration, eligibility recommendations, and implementation.

Evidence was selected according to its direct relevance to the clinical domains addressed in this review, including the cardiovascular burden of RSV infection, efficacy and effectiveness of adult RSV vaccines, cardiovascular outcomes following vaccination, safety and co-administration, and implementation within cardiovascular care pathways. Randomized trials, pragmatic studies, test-negative studies, observational cohorts, registry analyses, systematic reviews, trial registrations, and relevant regulatory or public-health documents were considered.

Because this was a narrative rather than a systematic review, study identification and selection were not performed independently in duplicate, no review protocol was prospectively registered, and no formal study-level risk-of-bias or certainty-of-evidence assessment was undertaken. Evidence was synthesized qualitatively according to study design and clinical question, with greater interpretative weight assigned to peer-reviewed randomized evidence and large, methodologically robust observational studies than to preliminary or non-peer-reviewed evidence.

## 4. RSV Infection as a Cardiopulmonary and Cardiovascular Trigger

**Figure 1:**
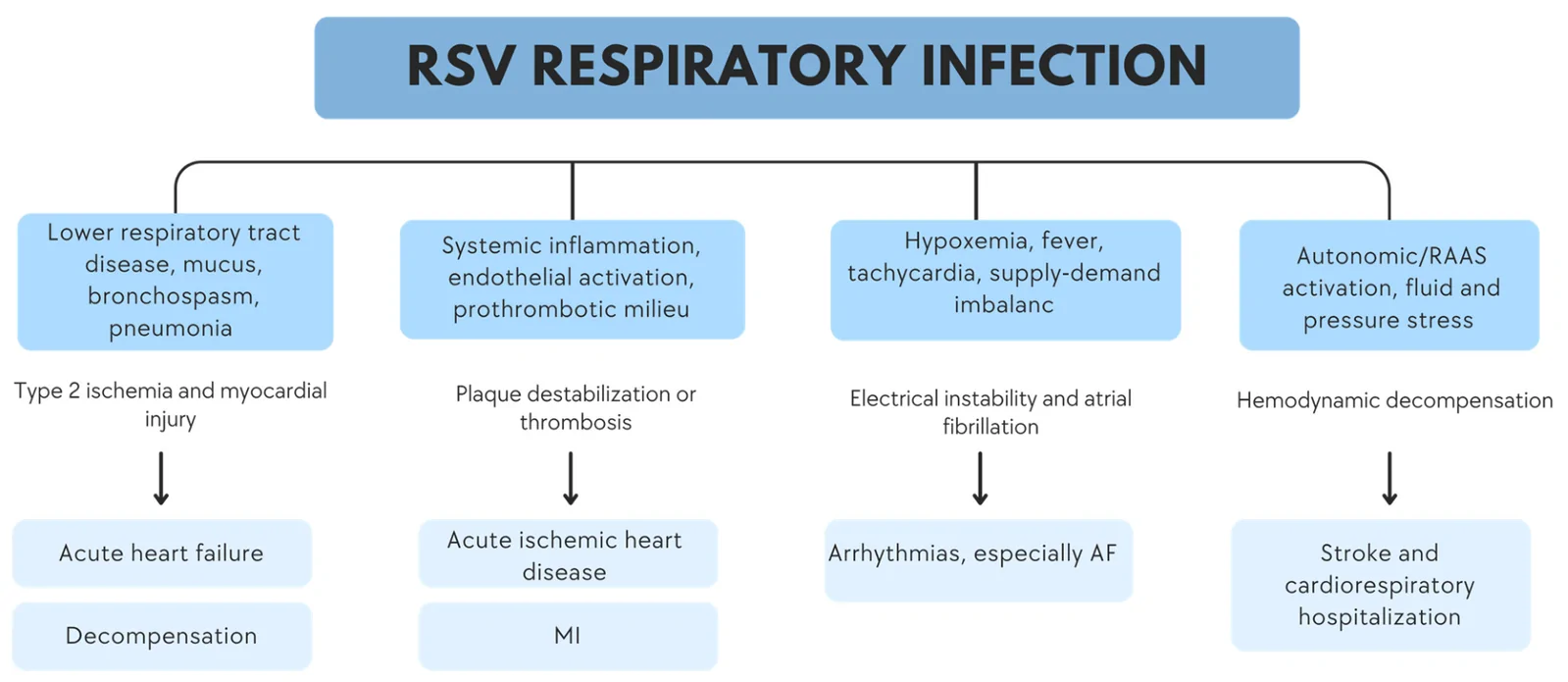
Potential mechanisms linking RSV infection to cardiovascular events. Abbreviations: AF, atrial fibrillation; MI, myocardial infarction; RAAS, renin–angiotensin–aldosterone system; RSV, respiratory syncytial virus.

RSV is an enveloped, non-segmented, negative-sense single-stranded RNA virus belonging to the genus *Orthopneumovirus* within the *Pneumoviridae* family. Its surface attachment (G) and fusion (F) glycoproteins mediate attachment to and entry into respiratory epithelial cells, while the prefusion F protein constitutes the principal antigenic target of currently available RSV vaccines. Viral replication, particularly in ciliated airway epithelial cells, induces epithelial injury, impaired mucociliary clearance, mucus hypersecretion, airway oedema, and local inflammatory responses, potentially resulting in lower respiratory tract obstruction and impaired gas exchange. In older adults, immunosenescence, chronic cardiopulmonary disease, and reduced physiological reserve increase the likelihood of severe infection. The resulting hypoxemia, systemic inflammation, sympathetic activation, and increased hemodynamic stress may precipitate heart failure decompensation, myocardial ischemia, or arrhythmias, although the relative contribution of these mechanisms remains incompletely understood [3,20,21,22]. These potential pathways are summarized in Figure 1.

Heart failure and coronary artery disease are important markers of susceptibility to severe RSV disease. In a US population-based surveillance analysis conducted between 2015 and 2017, the adjusted rate of RSV-associated hospitalization was 26.7 per 10,000 adults with congestive heart failure, compared with 3.3 per 10,000 among adults without heart failure, corresponding to a rate ratio of 8.1. The highest absolute hospitalization rate was observed among adults aged ≥65 years with heart failure [5]. In a separate prospective population-based study, RSV-associated hospitalization rates were 4.0- to 33.2-fold higher among adults with congestive heart failure and 3.7- to 7.0-fold higher among those with coronary artery disease than among adults without the corresponding condition [23]. A recent systematic review, meta-analysis, and modeling study extended these findings to younger adults: among those aged 50–64 years, the estimated hospitalization rate ratios were 10.5 for heart failure and 4.8 for coronary artery disease, while heart failure was associated with a rate ratio of 31.6 among adults aged 18–49 years, although these estimates were based on limited data and had wide confidence intervals [24].

Acute cardiac complications are also common during RSV-associated hospitalization. In a large cross-sectional study of 6248 adults aged ≥50 years hospitalized with laboratory-confirmed RSV, the weighted prevalence of an acute cardiac event was 22.4%. Events occurred in 33.0% of patients with underlying cardiovascular disease compared with 8.5% of those without cardiovascular disease, corresponding to an adjusted risk ratio of 3.51. Acute heart failure was the most frequent event, followed by acute ischemic heart disease, and cardiac events were associated with greater risks of intensive care unit admission and in-hospital death [2].

Temporal analyses provide additional support for RSV as an acute cardiovascular trigger, although the evidence remains observational. In a nationwide Danish self-controlled case-series analysis, RSV infection was associated with a marked increase in cardiovascular events during the 14 days following a positive test, particularly heart failure hospitalization and ischemic stroke [4]. In a separate self-controlled case-series analysis of 11,887 adults with RSV-related hospitalization, Liang et al. found increased risks of myocardial infarction, stroke, heart failure exacerbation, and arrhythmia, with the highest incidence rate ratios during the first one to two weeks and significant excess risks persisting beyond the acute phase [25]. Consistently, among 471 adults hospitalized with RSV, Sudnik et al. reported an 18.5-fold increase in cardiovascular-event incidence during the first 28 days compared with the six months before hospitalization; incidence remained 1.6-fold higher from day 29 to six months, and 44% of acute events occurred in patients without a previous history of the corresponding condition [26].

Cardiovascular risk may also extend over longer follow-up. In a nationwide Danish matched cohort, RSV infection was associated with 4.69 additional cardiovascular events per 100 infected individuals over one year, with the greatest absolute excess among hospitalized patients, older adults, and individuals with pre-existing cardiovascular disease [3]. A recent systematic review and meta-analysis by Montiel et al. estimated pooled absolute risks of 19.2% for any cardiac event and 15.7% for heart failure among hospitalized adults, although substantial heterogeneity and variation in outcome definitions should be considered [27].

Taken together, these observational findings indicate that RSV is associated with a disproportionate hospitalization burden among adults with cardiovascular disease and may precipitate both exacerbations of pre-existing conditions and first cardiovascular presentations. Nevertheless, they do not establish that the excess cardiovascular events associated with RSV infection are preventable through vaccination.

## 5. Benefits of RSV Vaccination in the General Older-Adult Population

The objective of this review is not to compare individual RSV vaccine products but to summarize the evidence supporting RSV vaccination as a prevention strategy in adults at increased cardiopulmonary and cardiovascular risk. Three principal adult RSV vaccine platforms are now relevant to clinical practice: an AS01E-adjuvanted RSV prefusion F protein vaccine (RSVPreF3 OA), a bivalent RSV prefusion F protein vaccine (RSVpreF), and an mRNA-based RSV prefusion F vaccine (mRNA-1345). Their pivotal trials enrolled large populations of adults, mainly aged 60 years or older, and demonstrated protection against RSV-associated lower respiratory tract disease or acute respiratory illness [8,9,10].

All three adult RSV vaccines have demonstrated favorable safety profiles and efficacy in preventing RSV-associated lower respiratory tract disease in their respective pivotal Phase III clinical trials. However, direct comparisons between vaccines should be avoided because efficacy estimates derive from studies with different designs, enrolled populations, clinical endpoint definitions, RSV circulation, case-accrual timing, and follow-up durations [8,9,10,11,12,28,29].

Duration of protection is now a central practical issue in adult RSV vaccination. Published peer-reviewed follow-up data are available for up to three RSV seasons for the adjuvanted RSVPreF3 vaccine and for two RSV seasons for the bivalent RSVpreF vaccine [11,12]. For mRNA-1345, the pivotal publication reported the initial efficacy analysis, while regulatory and public-health materials include longer follow-up analyses extending to approximately 18.8 months; these data should be described as longer-term follow-up rather than used for indirect product comparisons [10,28,29]. Across platforms, waning of efficacy over time has been observed, reinforcing the need to distinguish initial efficacy from durability and to avoid presenting single time-point estimates without their corresponding follow-up period.

Systematic review evidence supports the overall efficacy signal. A 2025 Cochrane review reported that, in four studies including 99,931 older adults, RSV prefusion vaccines reduced RSV-related respiratory illness and RSV-related acute illness, with high confidence for these respiratory outcomes. The review also noted low-confidence evidence suggesting little or no difference in serious unwanted effects or death, and importantly, that the adult studies did not report RSV-related hospitalization or intensive-care admission outcomes [13].

Hospitalization evidence has strengthened after the original pivotal efficacy trials. In the DAN-RSV pragmatic randomized trial of adults aged 60 years or older in Denmark, RSVpreF reduced hospitalization for RSV-related respiratory tract disease and RSV-related lower respiratory tract disease compared with no vaccine, with similar serious adverse-event incidence in the two groups [16]. The two-season effectiveness study by Surie et al. from the IVY Network showed that RSV vaccination was effective against RSV-associated hospitalization among adults aged 60 years or older, with stronger point estimates for same-season vaccination than for prior-season vaccination [18].

The general-population older-adult evidence therefore supports RSV vaccination as prevention of RSV disease and severe respiratory hospitalization. The inference that this will reduce some downstream cardiorespiratory destabilization is plausible, particularly in high-risk patients, but hard cardiovascular endpoints require direct evidence.

## 6. Co-Administration with Influenza and COVID-19 Vaccines

Current regulatory and public-health positions on RSV vaccine co-administration are broadly permissive, although recommendations remain product- and jurisdiction-specific [15,30,31]. In the United States, the Centers for Disease Control and Prevention permits RSV vaccines to be administered during the same visit as other adult vaccines, including influenza and COVID-19 vaccines [15]. In the European Union, concomitant administration should follow the product-specific Summary of Product Characteristics. For example, mRESVIA may be administered concomitantly with standard- or high-dose unadjuvanted seasonal influenza vaccines and an mRNA COVID-19 vaccine [30]. National implementation policies may nevertheless be more restrictive. Current UK guidance encourages co-administration of RSV and COVID-19 vaccines but does not recommend routinely scheduling Abrysvo with influenza vaccination because of possible attenuation of RSV- and influenza-specific immune responses [31]. However, same-day administration remains acceptable when immediate protection is required, or the individual is considered unlikely to return for a subsequent visit [31]. Therefore, clinicians should consult current national recommendations and the relevant product information rather than assuming a uniform class-wide approach [15,30,31].

Randomized co-administration studies have so far provided reassuring immunogenicity and short-term safety findings. In adults aged 65 years or older, concomitant administration of bivalent RSVpreF and seasonal inactivated influenza vaccine met the prespecified immunogenicity non-inferiority criteria and showed an acceptable safety and tolerability profile [32]. Similar findings were reported when adjuvanted RSVPreF3 OA was co-administered with an adjuvanted seasonal influenza vaccine [33]. A phase 3 study in adults aged 50 years or older found that mRNA-1345 could be co-administered with either a standard-dose inactivated influenza vaccine or an mRNA COVID-19 vaccine, with non-inferior immune responses and acceptable short-term safety [34]. A further randomized study in adults aged 65 years or older evaluated concomitant administration of RSVpreF and BNT162b2, with or without a quadrivalent influenza vaccine, and reported acceptable short-term safety and immunogenicity [35]. Across these studies, most solicited local and systemic reactions were mild or moderate and transient [32,33,34,35]. Nevertheless, same-day administration may increase the frequency of common reactions such as injection-site pain, fatigue, headache, myalgia, or fever [15,33]. Modest reductions in some antibody measurements have also been observed, although their clinical significance remains uncertain [15,31,32,33,34]. Furthermore, the available trials were primarily designed to assess immunogenicity and short-term safety and were not powered to detect rare adverse events or differences in clinical vaccine effectiveness [32,33,34,35].

For adults with stable heart failure or coronary artery disease, current guidance does not identify the cardiovascular diagnosis itself as a contraindication to co-administration [15,30,31]. Same-visit vaccination may be particularly advantageous in patients with multimorbidity, mobility limitations, or frailty by reducing the number of healthcare visits and missed vaccination opportunities, particularly when the likelihood of returning for another appointment is low [15,31]. Nevertheless, cardiovascular-specific evidence remains limited. Published co-administration trials predominantly enrolled healthy or medically stable adults and were not designed or powered to evaluate heart failure decompensation, acute coronary events, arrhythmias, MACE, cardiovascular mortality, or differences according to left ventricular ejection fraction, New York Heart Association functional class, recent acute coronary syndrome, or frailty severity [32,33,34,35]. Their findings should therefore not be directly extrapolated to patients with recently unstable cardiovascular disease or advanced frailty. In these patients, the choice between same-day and staggered administration should be individualized, balancing the need for timely protection and the likelihood of returning for another visit against anticipated reactogenicity and the potential value of attributing post-vaccination symptoms to a specific product [15,31]. Importantly, there is currently no evidence that co-administration either improves or worsens cardiovascular outcomes following RSV vaccination [32,33,34,35]. Dedicated pragmatic studies incorporating adjudicated cardiovascular endpoints and frailty-stratified analyses are needed.

## 7. Cardiovascular Outcomes Following RSV Vaccination: Randomized and Real-World Evidence

Evidence that RSV vaccination reduces cardiovascular events remains emerging and should be interpreted according to study design, endpoint definition, and statistical power. Adults with cardiovascular disease represent a clinically important population because of their high baseline risk of decompensation and hospitalization. Nevertheless, prevention of RSV disease and prevention of cardiovascular events are distinct clinical outcomes.

Randomized evidence is derived primarily from prespecified secondary analyses of DAN-RSV, a large pragmatic, open-label, individually randomized trial comparing bivalent RSVpreF vaccination with no vaccination in adults aged 60 years or older. RSVpreF vaccination significantly reduced all-cause cardiorespiratory hospitalization. In contrast, all-cause cardiovascular hospitalization was not significantly reduced, and no statistically significant between-group differences were demonstrated for myocardial infarction, heart failure hospitalization, atrial fibrillation, or stroke [17]. Importantly, the positive cardiorespiratory composite included respiratory as well as cardiovascular hospitalizations and therefore cannot be interpreted as evidence of a cardiovascular-specific vaccine effect. Moreover, the individual cardiovascular outcomes were exploratory, and the relatively low number of events limited statistical precision.

**Figure 2:**
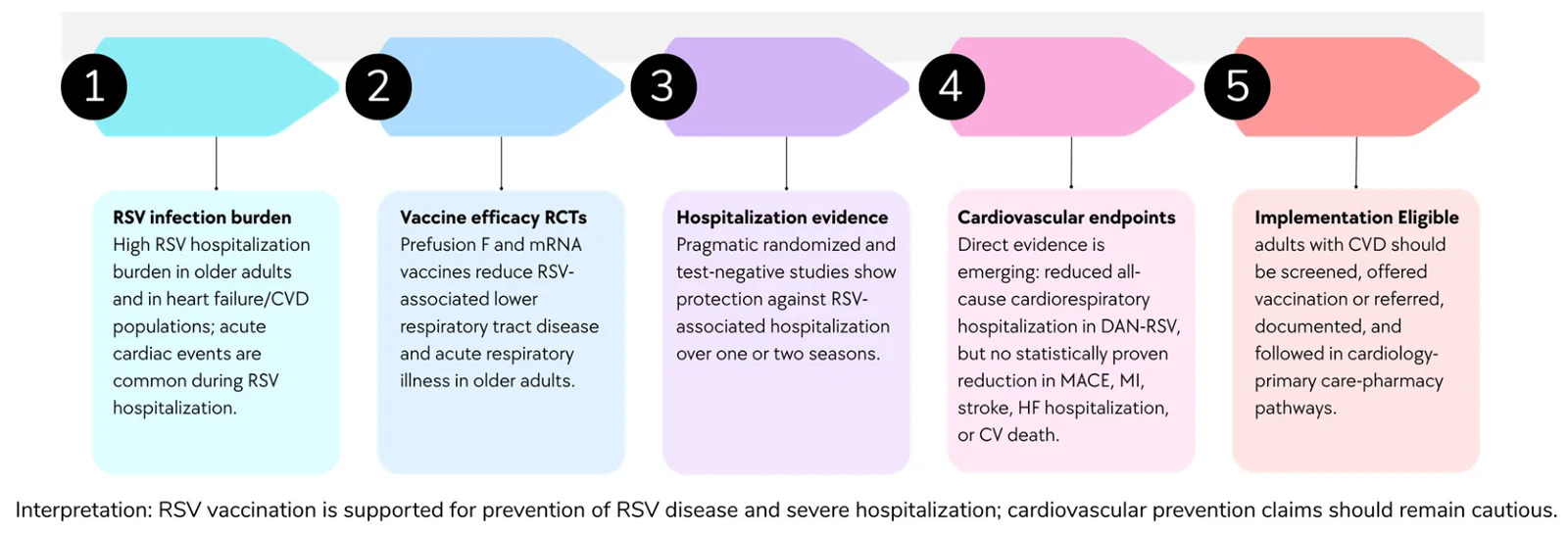
Evidence map for RSV vaccination and cardiovascular risk mitigation. The respiratory efficacy evidence is robust, hospitalization evidence is strengthening, and direct cardiovascular endpoint evidence is promising but not yet definitive. Abbreviations: CV, cardiovascular; CVD, cardiovascular disease; HF, heart failure; MACE, major adverse cardiovascular events; MI, myocardial infarction; RCT, randomized controlled trial; RSV, respiratory syncytial virus.

A further prespecified analysis stratified by atherosclerotic cardiovascular disease status found that vaccine effectiveness against RSV-related hospitalization endpoints was generally consistent among participants with and without pre-existing atherosclerotic cardiovascular disease (ASCVD). Although absolute cardiovascular event rates were higher among participants with ASCVD, vaccine-effectiveness estimates for MACE were imprecise and not statistically conclusive [19]. Collectively, these randomized findings support a potential overall cardiorespiratory benefit of vaccination but do not establish a reduction in MACE or cardiovascular mortality (Figure 2) (Table 1).

Accordingly, the established clinical rationale for RSV vaccination in eligible adults with cardiovascular disease remains the prevention of RSV disease and severe respiratory or cardiorespiratory hospitalization. A reduction in MACE or cardiovascular mortality is biologically plausible and increasingly supported by emerging evidence, but it has not yet been conclusively demonstrated and requires studies adequately powered for cardiovascular endpoints (Table 2).

## 8. Current Recommendations Relevant to Cardiovascular Practice

Clinical recommendations have evolved rapidly and remain jurisdiction- and time-specific. In the United States, current Centers for Disease Control and Prevention recommendations, following Advisory Committee on Immunization Practices guidance, support a single dose of any FDA-licensed RSV vaccine for all adults aged ≥75 years and for adults aged 50–74 years at increased risk of severe RSV disease. Qualifying risk factors include chronic cardiovascular disease, such as heart failure and coronary artery disease, whereas isolated hypertension alone is not included. These US eligibility criteria should not be extrapolated directly to other healthcare systems. For example, under the vaccination programme effective in England from 1 September 2026, a single dose is offered to adults aged ≥75 years, adults residing in or moving into care homes for older adults, and adults aged 65–74 years who are immunosuppressed or have chronic respiratory disease. Chronic cardiovascular disease alone is not currently listed as an eligibility criterion for the latter age group. Accordingly, vaccination assessment and clinical implementation pathways should follow current national recommendations, product-specific authorisations, and local delivery policies [15,31].

The RSV vaccine is not currently an annual vaccine. Eligible adults who have already received one dose of the adult RSV vaccine are not currently recommended to receive another dose, pending further data on the duration of protection and the revaccination strategy [15]. Vaccination is usually scheduled for late summer or early autumn in regions where RSV circulates during autumn and winter but may be offered on an opportunistic basis where clinically appropriate and in accordance with local regulations.

The 2025 European Society of Cardiology clinical consensus statement and the 2025 American College of Cardiology guidance both place adult immunization within cardiovascular care rather than treating it as a peripheral public-health issue [37,38]. For RSV, the cardiology implication is pragmatic: cardiology teams should identify eligible patients, discuss benefits and uncertainties, coordinate delivery or referral, and document vaccination status (Table 3).

## 9. Vaccine Formulations, Dosing, Duration, and Safety

Available adult RSV vaccine products differ in platform, regulatory indication, follow-up duration, and maturity of durability evidence. RSVPreF3 OA is an adjuvanted prefusion F protein vaccine; RSVpreF is a bivalent prefusion F protein vaccine; and mRNA-1345 encodes stabilized prefusion F protein. Current United States clinical guidance does not express a product preference among FDA-licensed adult RSV vaccines for recommended adults [15]. Product selection should therefore follow national or regional recommendations, availability, contraindications, prior vaccine status, and shared decision-making rather than cross-trial efficacy ranking (Table 4).

The current adult schedule is a single dose, not annual revaccination. This differs from influenza vaccination and is a critical implementation point because duplicate seasonal prompts can generate unnecessary repeat vaccination unless prior status is checked. Ongoing surveillance and follow-up studies will determine whether and when revaccination is needed; available durability data should be interpreted by product, time since vaccination, population risk, and outcome definition [11,12,15,18,28,29].

Safety should be discussed transparently. In pivotal trials, local and systemic reactogenicity was generally more common after vaccination than placebo, but serious adverse events were broadly similar between vaccine and control groups [8,9,10,13]. Post-licensure surveillance has evaluated rare neurologic events, including Guillain-Barre syndrome, particularly after protein-subunit vaccines. These rare signals require monitoring and shared decision-making in individual patients, but current recommendations continue to support vaccination in eligible adults at risk of severe RSV [14,15].

Coadministration with other indicated adult vaccines may reduce missed vaccination opportunities and can be considered in accordance with national recommendations, product information, and patient preference. Because evidence on reactogenicity and immunogenicity continues to evolve, decisions should be individualized according to the vaccines involved and the clinical context. Although reactogenicity and immunogenicity data continue to evolve, routine coadministration is widely supported by local guidelines across Western countries. Clinical decisions should prioritize minimizing the risk of missed vaccinations that often occurs when appointments are separated, in alignment with standard guidelines, product information, and patient preference.

## 10. Implementation in Cardiovascular Care

Adult RSV vaccination will not reduce RSV-related hospitalization or subsequent cardiorespiratory destabilization if eligible patients are not identified. Cardiologists do not need to become vaccination specialists, but they should have clear pathways for screening, referral, documentation, and communication.

**Figure 3:**
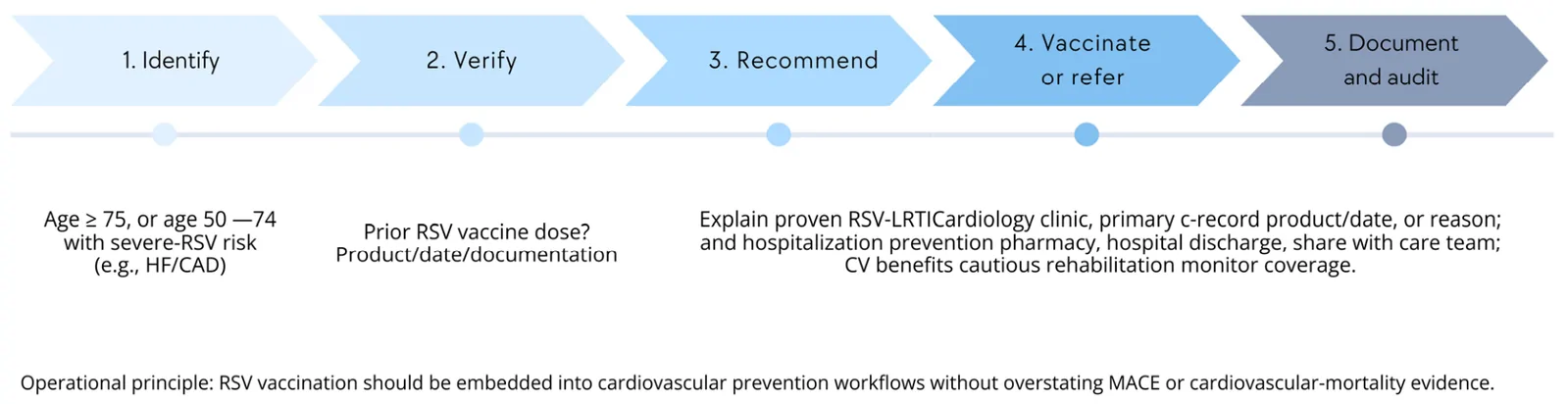
Practical pathway for implementing RSV vaccination in cardiovascular care. The pathway emphasizes eligibility assessment, prior-dose verification, balanced counseling, vaccination or referral, documentation, and audit. It also preserves scientific restraint by framing RSV vaccination as cardiopulmonary risk mitigation rather than proven MACE therapy. Abbreviations: CAD, coronary artery disease; CV, cardiovascular; HF, heart failure; MACE, major adverse cardiovascular events; RSV, respiratory syncytial virus.

A survey of cardiovascular professionals identified a gap between recognizing vaccination as a preventive strategy and recommending it routinely, particularly among residents. These findings highlight the need for practical implementation pathways within cardiovascular care (Figure 3).

A cardiovascular implementation pathway should include four steps. First, identify patients who are clearly eligible by age alone, especially those aged 75 years or older. Second, identify adults aged 50 to 74 years with qualifying risk conditions, including chronic cardiovascular disease such as heart failure or coronary artery disease. Third, verify whether a prior adult RSV vaccine dose has already been administered. Fourth, offer vaccination directly if the clinical setting permits, or refer to primary care or pharmacy with explicit documentation.

Heart failure pathways deserve special attention. Acute heart failure admissions and post-discharge visits are frequent missed opportunities. As a fundamental step, the cardiovascular patient’s medical history (anamnesis) should routinely document whether they have received the RSV vaccine and the date of administration. Furthermore, for unvaccinated patients, the recommendation to vaccinate should be explicitly added to hospital discharge reports, alongside incorporating vaccination status checks into nurse-led heart failure calls, device-clinic workflows, and cardiac rehabilitation intake. Because RSV vaccination is currently not annual, the workflow should focus on one-time status verification rather than yearly re-ordering.

For patients with acute coronary syndrome, stable coronary artery disease, valvular disease, or atrial fibrillation, RSV vaccination should be considered according to age and risk criteria rather than as a treatment for the acute event itself. Vaccination should complement, not replace, standard guideline-directed cardiovascular therapies (Table 5).

## 11. Practical Barriers to Implementation Within Cardiology Services

Successful integration of RSV vaccination into cardiovascular care requires more than identifying eligible patients. Cardiology teams may need targeted training on eligibility criteria, product-specific recommendations, co-administration, relevant precautions, expected reactogenicity, and communication of the established and still-uncertain benefits of vaccination. Clear allocation of responsibilities is also essential, including who assesses eligibility, recommends or administers the vaccine, documents the intervention, and follows up patients who defer vaccination. Access may be unequal when vaccine availability, reimbursement, local supply, mobility limitations, socioeconomic factors, or reliance on referral to primary care or community pharmacies create additional barriers, particularly for frail or socially vulnerable adults. Whenever feasible, on-site vaccination or direct referral pathways may reduce missed opportunities. Electronic health record workflows should include structured fields for vaccine product, administration date, co-administered vaccines, and reason for non-vaccination, together with interoperability across cardiology, primary care, pharmacy, and regional immunization registries. Automated eligibility prompts, duplicate-dose alerts, and audit indicators for assessment, recommendation, uptake, and equity may further support implementation. Local pathways should therefore be adapted to available staffing, procurement, storage, reimbursement, and digital infrastructure.

## 12. Limitations of the Available Evidence

Several limitations should be considered. Observational estimates may be affected by incomplete or differential RSV testing, diagnostic coding, healthcare access, and systematic differences between tested, hospitalized, vaccinated, and unvaccinated populations, leaving potential for selection bias, misclassification, and residual confounding. In addition, pivotal vaccine trials were primarily designed to assess respiratory efficacy and safety and were not specifically powered to evaluate cardiovascular hospitalization, individual cardiovascular events, MACE, cardiovascular mortality, or uncommon cardiac adverse events.

The generalizability of the available evidence across geographic settings also remains uncertain. Most randomized and real-world evidence derives from a limited number of regions and RSV seasons, whereas RSV seasonality, circulating subtypes or strains, healthcare-seeking behavior, diagnostic practices, baseline cardiovascular risk, vaccine uptake, and national vaccination policies vary across countries. These epidemiological and programmatic differences may influence both the absolute burden of preventable disease and the observed effectiveness of vaccination, limiting direct extrapolation to settings that are underrepresented in current studies.

Evidence is also sparse for adults younger than 50 years with high-risk cardiovascular conditions, who have been underrepresented in the available efficacy and cardiovascular-outcome studies. Consequently, findings obtained predominantly in older adult populations should not be assumed to apply directly to younger but clinically vulnerable individuals, and dedicated studies in this population are needed.

Interpretation of cardiovascular endpoints requires further caution because event definitions, adjudication procedures, follow-up duration, and the presumed causal relationship between RSV and individual outcomes vary across studies. Composite outcomes may also combine events with differing biological plausibility. Furthermore, standardized protocols for long-term cardiac adverse-event surveillance following RSV vaccination are lacking. Differences in active versus passive event ascertainment, surveillance windows, endpoint adjudication, and adjustment for background cardiovascular risk limit comparisons across studies and the ability to detect rare, delayed, or product-specific safety signals. Harmonized prospective surveillance protocols incorporating prespecified cardiac endpoints, standardized adjudication, appropriate comparator groups, and sufficiently long follow-up are therefore needed.

Evidence regarding durability of protection, revaccination, and product-specific performance remains evolving, while cross-trial comparisons are constrained by differences in study design, follow-up, endpoint definitions, participant characteristics, and RSV circulation. Finally, our study is a narrative rather than a systematic review. Study identification, inclusion, and emphasis were influenced by author judgement and may therefore be affected by selection, publication, citation, and availability bias. Consequently, the conclusions should be interpreted as a clinically oriented qualitative synthesis rather than as an exhaustive, systematically selected, or formally graded estimate of the available evidence.

## 13. Conclusions

RSV is an important cause of severe respiratory disease and cardiovascular destabilization in older adults and those with chronic heart disease. Adult RSV vaccines effectively prevent RSV-associated respiratory illness and hospitalization, but evidence for reducing specific cardiovascular events remains limited.

Current data support RSV vaccination as a strategy to reduce severe cardiopulmonary disease rather than as a proven intervention for preventing MACE, myocardial infarction, stroke, heart failure hospitalization, or cardiovascular death. Implementation should therefore focus on identifying eligible adults, confirming vaccination status, and integrating vaccination into routine cardiovascular care alongside established therapies.

## Figures and Tables

**Table 1:** Key evidence on RSV infection, differential disease burden according to underlying cardiovascular disease, and outcomes following RSV vaccination.

Study/Source	Population/Design	Main Result	Interpretation for Adults with Heart Disease
Cardiovascular and cardiorespiratory outcomes associated with RSV infection			
Woodruff et al. [2]	6248 adults aged ≥50 years hospitalised with laboratory-confirmed RSV	The weighted prevalence of an acute cardiac event was 22.4%. Events occurred in 33.0% of patients with underlying CVD compared with 8.5% of those without CVD.	Acute cardiac complications are common during severe RSV disease, particularly in patients with pre-existing CVD, but may also occur in those without previously documented CVD.
Hviid et al. [3]	Danish matched cohort of adults aged ≥45 years with laboratory-confirmed RSV infection	RSV infection was associated with 4.69 additional cardiovascular events per 100 infected adults over 1 year; the absolute excess was greatest in patients with pre-existing CVD.	Suggests that cardiovascular risk may extend beyond the acute infectious episode.
Liang et al. [25]	US self-controlled case-series analysis of 11,887 adults with RSV-related hospitalisation	Risks of myocardial infarction, stroke, heart failure exacerbation, and arrhythmia were highest during the first 1–2 weeks, with excess risk persisting beyond the acute phase for some outcomes.	Supports RSV as a potential acute and post-acute cardiovascular trigger; it is not evidence of vaccine effectiveness.
Sudnik et al. [26]	Retrospective cohort of 471 adults hospitalised with RSV	Cardiovascular-event incidence increased 18.5-fold during the first 28 days and remained 1.6-fold higher from day 29 to 6 months; 44% of events occurred without a previous history of the corresponding condition.	RSV hospitalisation may precipitate both exacerbations of established CVD and first cardiovascular presentations.
RSV disease burden among adults with and without underlying cardiovascular disease			
Kujawski et al. [5]	US population-based surveillance of adults with and without congestive heart failure, 2015–2017	The adjusted RSV-associated hospitalisation rate was 26.7 per 10,000 adults with heart failure and 3.3 per 10,000 without heart failure, corresponding to a rate ratio of 8.1.	Heart failure identifies a population with a markedly increased absolute and relative RSV hospitalisation burden.
Branche et al. [23]	Prospective US population-based surveillance, 2017–2020	RSV-associated hospitalisation rates were 4.0- to 33.2-fold higher with heart failure and 3.7- to 7.0-fold higher with coronary artery disease than without the corresponding condition.	Demonstrates a consistent differential burden according to underlying CVD across age groups and surveillance settings.
Li et al. [24]	Systematic review, meta-analysis, and modelling study in adults aged 18–64 years	Among adults aged 50–64 years, estimated hospitalisation rate ratios were 10.5 for heart failure and 4.8 for coronary artery disease; the estimated ratio for heart failure was 31.6 among those aged 18–49 years.	Suggests substantial disease burden in younger high-risk adults, although estimates were based on limited data and had wide confidence intervals.
Outcomes following RSV vaccination			
Pivotal adult vaccine trials: AReSVi-006, RENOIR, and ConquerRSV [8,9,10,11,12]	Phase 3 RCTs of RSVPreF3 OA, RSVpreF, and mRNA-1345 in adults aged ≥60 years	The three vaccine platforms demonstrated efficacy against RSV-associated lower respiratory tract disease or illness; follow-up and durability evidence differ by product and trial.	Establishes prevention of RSV respiratory disease; these trials were not designed to demonstrate reductions in specific cardiovascular events.
DAN-RSV and prespecified cardiovascular analyses [16,17,19]	Pragmatic randomised trial of 131,276 adults aged ≥60 years, including analyses by ASCVD status	Vaccination reduced RSV-related and all-cause cardiorespiratory hospitalisation. All-cause cardiovascular hospitalisation and individual cardiovascular endpoints were not significantly reduced; MACE estimates were imprecise.	Supports prevention of severe RSV and an overall cardiorespiratory benefit, but does not establish reductions in MACE or cardiovascular mortality.
Surie et al. [18]	US test-negative study over two RSV seasons in adults aged ≥60 years	RSV vaccination was effective against RSV-associated hospitalisation, with lower point estimates for prior-season than for same-season vaccination.	Supports effectiveness against severe RSV hospitalisation and highlights the potential relevance of time since vaccination.
CLEAR-VE/Singer et al. [36]	US retrospective claims-based cohort of adults aged ≥60 years receiving adjuvanted RSVPreF3 or remaining unvaccinated	VE was 75.6% against RSV-related hospitalisation overall and 72.2% among adults with CVD. Among adults with CVD, estimated VE against RSV-related MACE was 63.1% when mortality was excluded.	Provides supportive real-world evidence, but the non-randomised, claims-based, product-specific design remains susceptible to residual confounding and outcome misclassification. Cardiovascular findings remain hypothesis-generating.

Abbreviations: ASCVD, atherosclerotic cardiovascular disease; CVD, cardiovascular disease; MACE, major adverse cardiovascular events; RCT, randomised controlled trial; RSV, respiratory syncytial virus; VE, vaccine effectiveness.

**Table 2:** Evidence boundaries for interpreting respiratory, cardiorespiratory, and cardiovascular outcomes following adult RSV vaccination.

Clinical Statement	Current Evidence Status	Recommended Wording	Interpretative Boundary
Adult RSV vaccination reduces the risk of RSV-associated LRTD or ARI.	Established. Supported by pivotal randomised phase 3 trials across currently available adult RSV vaccine platforms.	“Adult RSV vaccination reduces the risk of RSV-associated lower respiratory tract disease and acute respiratory illness in eligible adults.”	Protection against respiratory disease should not be interpreted as evidence of a direct reduction in cardiovascular events.
Adult RSV vaccination reduces RSV-associated hospitalisation.	Supported. Evidence derives from pragmatic randomised studies and multiple test-negative and real-world effectiveness analyses.	“Current evidence supports a clinically relevant reduction in RSV-associated hospitalisation among eligible older and high-risk adults.”	This conclusion applies specifically to RSV-related hospitalisation and should not be extrapolated to all-cause or cardiovascular hospitalisation unless those endpoints were evaluated directly.
RSV vaccination reduces all-cause cardiorespiratory hospitalisation.	Trial-specific randomised signal. A prespecified analysis of DAN-RSV demonstrated a statistically significant reduction with bivalent RSVpreF vaccination.	“In DAN-RSV, bivalent RSVpreF vaccination reduced all-cause cardiorespiratory hospitalisation in adults aged ≥60 years.”	The result is product- and study-specific. Because the composite included both respiratory and cardiovascular hospitalisations, it does not demonstrate a cardiovascular-specific vaccine effect and should not be generalised to all RSV vaccines.
RSV vaccination reduces all-cause cardiovascular hospitalisation or individual cardiovascular events.	Not demonstrated in randomised evidence. DAN-RSV did not show statistically significant reductions in all-cause cardiovascular hospitalisation, myocardial infarction, heart failure hospitalisation, atrial fibrillation, stroke, cardiovascular death, or MACE.	“A reduction in cardiovascular hospitalisation or specific cardiovascular events has not yet been demonstrated in randomised trials.”	Respiratory or cardiorespiratory benefits must not be presented as evidence of direct cardiovascular risk reduction.
RSV vaccination reduces MACE, myocardial infarction, stroke, or cardiovascular mortality.	Unproven. Available randomised analyses are neutral or statistically imprecise, while favourable real-world associations remain observational and susceptible to residual confounding and outcome misclassification.	“Cardiovascular-event reduction remains a biologically plausible and clinically important hypothesis, but it has not been established as a vaccine benefit.”	Observational signals should be considered hypothesis-generating and must not support claims of cardioprotection. Endpoint-driven randomised trials with adjudicated cardiovascular outcomes are required.

Abbreviations: ARI, acute respiratory illness; LRTD, lower respiratory tract disease; MACE, major adverse cardiovascular events; RSV, respiratory syncytial virus.

**Table 3:** Recommendation documents and cardiology-specific implications for RSV vaccination.

Source	Target Population	Vaccine/Dosing Implication	Cardiology-Specific Implementation Implication
CDC/ACIP adult guidance [14,15]	All adults aged ≥75 years; adults aged 50–74 years at increased risk of severe RSV, including chronic cardiovascular disease such as heart failure or coronary artery disease.	Single adult RSV vaccine dose; no product preference among FDA-licensed adult RSV vaccines where eligible; not currently annual.	Cardiology visits should include eligibility screening, previous-dose verification, and vaccination or referral.
FDA/EMA regulatory sources [39,40]	Licensed indications vary by jurisdiction, age, and risk group.	Product labels and regional authorizations may differ from public-health recommendations.	Use local formulary and national/regional recommendations before ordering or advising product-specific vaccination.
ESC consensus statement [37]	Patients with cardiovascular disease and high-risk profiles.	Vaccination recognized as part of infection-related cardiovascular risk prevention.	Supports embedding immunization assessment into cardiovascular prevention pathways.
ACC concise clinical guidance [38]	Adults with cardiovascular disease encountered in inpatient and outpatient cardiovascular care.	Adult immunization is framed as a routine care opportunity; implementation depends on eligibility, contraindications, and local policy.	Supports quality-improvement workflows in cardiology, heart failure, and transitions of care.

Abbreviations: ACC, American College of Cardiology; CDC, Centers for Disease Control and Prevention; EMA, European Medicines Agency; ESC, Euroepan Society of Cardiology; FDA, Food and Drug Administration; RSV, respiratory syncytial virus.

**Table 4:** Adult RSV vaccine platforms and practical cardiology interpretation.

Platform/Product Examples	Target Antigen	Pivotal and Durability Evidence	Current Practical Points	Cardiology Interpretation
Adjuvanted RSVPreF3 OA (Arexvy, GSK)	Stabilized prefusion F protein with AS01E adjuvant of VRSA	AReSVi-006 demonstrated efficacy against RSV-related LRTD in adults ≥ 60 years; peer-reviewed follow-up supports efficacy across three RSV seasons (median follow-up approximately 30.6 months) [8,11].	Use according to national recommendations and local label. EMA and FDA/regional authorizations and public-health recommendations may differ by age and risk group.	Useful option for eligible older or high-risk adults; product-specific durability evidence is mature, but revaccination strategy remains a policy question.
Bivalent RSVpreF (Abrysvo, Pfizer)	Prefusion F proteins from RSV A and RSV B	RENOIR demonstrated efficacy against RSV-associated LRTI in adults ≥ 60 years; peer-reviewed follow-up is available across two RSV seasons (approximately 16.4 months disease surveillance in reported analyses) [9,12].	Single adult dose where recommended; also has maternal-infant indication in many jurisdictions.	Especially relevant because DAN-RSV directly informs hospitalization and cardiorespiratory outcomes in older adults; efficacy estimates should not be compared directly with other trials.
mRNA-1345 (mResvia, Moderna)	mRNA encoding stabilized prefusion F protein of VRSA	ConquerRSV demonstrated efficacy against RSV-associated LRTD in adults ≥ 60 years in the pivotal publication; longer follow-up analyses up to approximately 18.8 months are described in regulatory/public-health materials [10,28,29].	FDA-approved for adults ≥ 60 years and, in some jurisdictions, for younger adults at increased risk; local recommendations should be checked before use.	Adds a non-protein-subunit platform; longer-term estimates should be interpreted with their follow-up period and source, not as direct comparative product evidence.

Abbreviations: EMA, European Medicines Agency; FDA, Food and Drug Administration; LRTD, lower respiratory tract disease; RSV, respiratory syncytial virus. Direct comparisons between vaccine products are inappropriate because the pivotal and follow-up studies differed in design, enrolled population, endpoint definitions, RSV seasonality/circulation, case accrual, and duration of follow-up. The table summarizes the evidence base for clinical interpretation, not comparative ranking.

**Table 5:** Practical implementation pathways for RSV vaccination in cardiovascular care.

Care Setting	Trigger Point	Action	Operational Caution
Acute coronary syndrome discharge	Medication reconciliation, discharge checklist, early post-discharge visit.	Assess age/risk eligibility; verify prior RSV dose; refer or vaccinate if feasible and clinically stable.	Avoid presenting RSV vaccination as proven post-MI MACE therapy; frame as prevention of severe RSV illness in eligible adults.
Heart failure discharge	Admission for acute or decompensated HF; discharge planning; early HF clinic follow-up.	Prioritize because HF is a high-risk RSV phenotype; include RSV status in discharge bundle.	Coordinate with primary care/pharmacy to avoid missed one-time vaccination.
Outpatient cardiology	Annual cardiovascular review, pre-winter visits, device clinics, anticoagulation visits.	Prompt eligibility screening for adults ≥ 75 and at-risk adults 50–74.	Document product/date or reason not given.
Cardiac rehabilitation	Program intake and seasonal sessions.	Use standardized vaccine-status questions and referral templates.	Rehabilitation teams can close preventive-care gaps during repeated patient contacts.
Primary care	Chronic disease review and adult immunization visit.	Apply national/regional recommendations and ensure record integration.	Primary care should confirm cardiology risk diagnoses when needed.
Pharmacy-based pathways	Walk-in vaccination, seasonal campaigns, referral from cardiology/primary care.	Check eligibility and prior dose; provide product/date documentation to the medical record when possible.	Pharmacy access may improve uptake in ambulatory adults.
Long-term care	Resident admission, pre-season campaign, outbreak-preparedness review.	Identify eligible residents and coordinate consent, vaccination, and documentation.	High-risk communal setting; integrate with influenza and COVID-19 prevention planning while respecting RSV one-dose status.

Abbreviations: HF, heart failure; MACE, major adverse cardiovascular events; MI, myocardial infarction; RSV, respiratory syncytial virus.

## Data Availability

The original contributions presented in this study are included in the article. Further inquiries can be directed to the corresponding authors.

## Abbreviations

The following abbreviations are used in this manuscript:

ASCVD	Atherosclerotic cardiovascular disease
MACE	Major adverse cardiac events
RSV	Respiratory syncytial virus
